# Neurobrucellosis with intramedullary spinal cord involvement: A case report

**Published:** 2018-07-06

**Authors:** Vida Mohammadzadeh, Samira Akbarieh, Seyed Abdolreza Ghoreishi, Manizheh Jozpanahi

**Affiliations:** 1 Department of Neurology, Vali-e-Asr Hospital, Zanjan University of Medical Sciences, Zanjan, Iran; 2 Department of Infectious Diseases, Vali-e-Asr Hospital, Zanjan University of Medical Sciences, Zanjan, Iran

**Keywords:** Brucellosis, Neurobrucellosis, Spinal Cord Involvement, Intramedullary

Human brucellosis is a multisystem zoonotic infection in many developing countries. Brucellosis may present with variable clinical manifestations.^1^ Neurobrucellosis is the second most prevalent and debilitating feature of systemic brucellosis which presents as meningoencephalitis, radiculitis, myelitis, peripheral/cranial neuropathies, and psychiatric manifestations. The incidence of the neurobrucellosis has been reported 0.5%-25% in different studies. Although the involvement of central nervous system (CNS) is not very common, but it should be particularly considered because of its severity and morbidity.^2^

In this case report, we present a 52-year-old man who was admitted to our hospital because of progressive motor weakness in his legs, and urinary incontinence while showed no clinical-radiologic correlation.

He had been diagnosed with Brucella due to fever and joint manifestation. The triple therapy of doxycycline, ciprofloxacin, and rifampicin had been started 20 days before his admission date. Because of appearance of rifampicin side effects, this drug stopped, and the patient discharged with doxycycline and ciprofloxacin. Medical history confirmed that he had been consumed non-pasteurized cheese. During his medical treatment for brucellosis, a progressive loss of strength was developed in his lower extremities during two weeks. In addition, gradually gait disturbance, anorexia, vomiting, urinary incontinence, and disability in daily activities were added. On admission, the patient was completely conscious, cooperative, and oriented. The vital signs were within the normal limit. In his respiratory and cardiovascular examination, there was no specific abnormal findings with symmetric and normal peripheral pulses. There were no meningeal signs, too. In CNS examination, the cranial nerves and upper limb examination were normal. Strength loss with absent deep tendon reflexes (DTR) in the lower extremities, mute plantar reflexes, negative abdominal cutaneous reflexes (ACR), superficial sensory deficit at the level of T7, and a decreased sense of vibration were observed in his examinations. His symptoms progressed rapidly, so that he became paraplegic at the third day of hospitalization. We performed magnetic resonance imaging (MRI), and only mild spondylodiscitis at T11-T12 level with no compression effect on spinal cord or enhancement were revealed (Figure 1). The cerebrospinal fluid (CSF) analysis showed clear and colorless material with elevated protein level (1080 mg/dl, normal value: 15-45), lymphocytic pleocytosis, and low sugar value (28 mg/dl; normal value: 40-70). He had positive Wright test in both CSF and serum samples. Tuberculosis (TB) was excluded with negative polymerase chain reactions (PCR) for Mycobacterium tuberculosis (MTB). Serologic evaluations of other possible infections such as human immunodeficiency virus (HIV) were found negative. He had positive CSF and serum Wright agglutination with a titer of 1/80 and 1/160, respectively. The TB PCR in CSF was negative. Four drug regimens including doxycycline (200 mg/day), gentamicin (240 mg/day), intravenous ciprofloxacin (800 mg/day), and trimethoprim/sulfametoxazol (2400/480 mg/day) were administered. The doxycycline discontinued when the medical condition of patient became worse, so three drugs regimen continued. 

**Figure 1:**
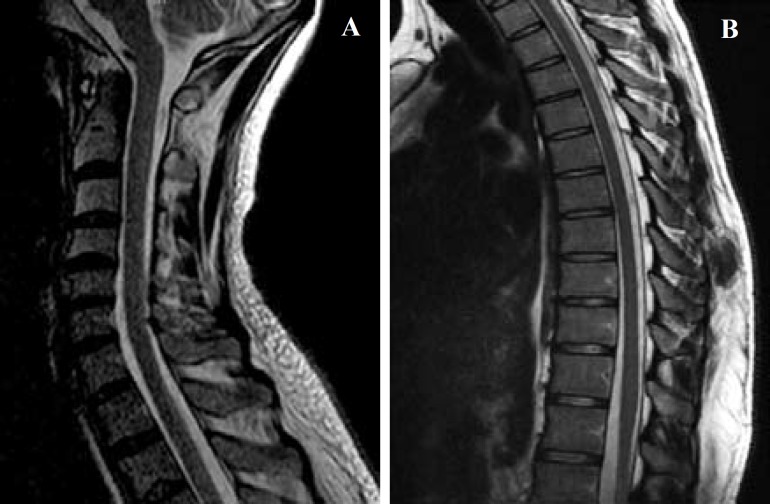
The images of sagital T2-weighted magnetic resonance imaging (MRI) of cervical (A) and thoracolumbar spine (B)

However, the patient unfortunately died two weeks after his admission due to gastrointestinal complications such as ileus and subsequent intestinal perforation, in spite of his treatment with neostigmine and other medications.

The spinal form of brucellosis is extremely rare, and can occur due to the compression of abscess, granuloma, or spinal root involvement.^3^ Prognosis of the neurobrucellosis in the encephalic or spinal cord involvement is poor, and mortality is not negligible. Here, we reported a case of neurobrucellosis presenting with myelitis signs and symptoms, which had no extramedullary involvement in his MRI. The distinctive prevalence of spinal form of the neurobrucellosis with normal spinal MRI is not well known.^4^ Based on our last knowledge, only one case has been reported which had intramedullary spinal cord brucellar involvement with normal MRI.^5^ We concluded that transverse myelitis has been occurred in our case due to brucellosis regarding to sensory deficit at the level of T7, paraplegia, sphincteric disturbance, and positive Wright/2-mercaptoethanol (2ME) tests in CSF analysis and rule out other suspected causes of paraplegia. Hence, it is important to keep in mind the probability of neurobrucellosis in patients with uncommon neurologic symptoms in endemic regions. 

## Notes:

**How to cite this article:** Mohammadzadeh V, Akbarieh S, Ghoreishi SA, Jozpanahi M. Neurobrucellosis with intramedullary spinal cord involvement: A case report. Iran J Neurol 2018; 17(3): 152-3.
